# Monitoring Traffic Information with a Developed Acceleration Sensing Node

**DOI:** 10.3390/s17122817

**Published:** 2017-12-05

**Authors:** Zhoujing Ye, Linbing Wang, Wen Xu, Zhifei Gao, Guannan Yan

**Affiliations:** 1 National Center for Materials Service Safety, University of Science and Technology Beijing, Beijing 100083, China; yezhoujing@126.com (Z.Y.); b20160405@xs.ustb.edu.cn (W.X.); b20160403@xs.ustb.edu.cn (Z.G.); b20150406@xs.ustb.edu.cn (G.Y.); 2 Joint USTB-Virginia Tech Lab on Multifunctional Materials, USTB, Beijing 100083, China & Virginia Tech, Blacksburg, VA 24061, USA

**Keywords:** traffic information monitoring, low energy consumption, acceleration sensing node, pavement vibration

## Abstract

In this paper, an acceleration sensing node for pavement vibration was developed to monitor traffic information, including vehicle speed, vehicle types, and traffic flow, where a hardware design with low energy consumption and node encapsulation could be accomplished. The service performance of the sensing node was evaluated, by methods including waterproof test, compression test, sensing performance analysis, and comparison test. The results demonstrate that the sensing node is low in energy consumption, high in strength, IPX8 waterproof, and high in sensitivity and resolution. These characteristics can be applied to practical road environments. Two sensing nodes were spaced apart in the direction of travelling. In the experiment, three types of vehicles passed by the monitoring points at several different speeds and values of *d* (the distance between the sensor and the nearest tire center line). Based on cross-correlation with kernel pre-smoothing, a calculation method was applied to process the raw data. New algorithms for traffic flow, speed, and axle length were proposed. Finally, the effects of vehicle speed, vehicle weight, and *d* value on acceleration amplitude were statistically evaluated. It was found that the acceleration sensing node can be used for traffic flow, vehicle speed, and other types of monitoring.

## 1. Introduction

Road information monitoring is a very important technique to ensure long lifespan of roads and traffic safety. For road information monitoring, the existing approaches can be categorized into non-intrusive and intrusive techniques. Conventionally, non-intrusive detection technologies include cameras, radar systems, light barriers, and pneumatic tubes, which are often susceptible to weather disruptions and vandalism [1,2,3]. The traditional intrusive technologies include induction loop detectors, pressure cell, deflect meter, strain gauge, thermocouple, moisture sensor, and fiber-optic sensors, which can often be rather costly for installation and suffer from a low survival rate [4,5,6,7]. Moreover, it is a very challenging and costly task to manage the huge amounts of data generated by a dense array of wired sensors [8,9,10].

More recently, accelerometer technologies have been substantially improved in terms of sensitivity, frequency response, physical size, and power consumption [11,12,13,14,15]. Due to these advantages, accelerometers are considered to be ideal candidates to monitor the pavement for wide-area instrumentation [16,17]. Under the vehicle load, an acceleration sensor with high precision can be applied to monitor the vibration of pavement. Yu et al. measured the vertical acceleration of pavement using an ICP (Integrated Circuit Piezoelectric) accelerometer with sensitivity of 100 mV/g. They introduced pavement evaluation based on measurements of the vertical acceleration of a driving vehicle [18]. Hostettler et al. used a three-axis piezoelectric accelerometer to measure and estimate vehicle parameters based on road surface vibrations. They proposed a model-based approach for vehicle counting and axle estimation based on the local energy estimation [19,20]. Arraigada et al. measured pavement deflections due to traffic loads by using inertial sensors. Deflections calculated from acceleration showed a reasonable qualitative correlation to those measured by magnetostrictive deflectometers [21]. Levenberg estimated vehicle speed and pavement layer properties with embedded single-axis high-end piezoelectric accelerometers [22,23]. Unfortunately, the commercial piezoelectric acceleration sensor is often required to be equipped with an adapter and a data acquisition device [22]. In practice, there are a lot of disadvantages, such as high energy consumption, large size, high cost, and non-real-time data processing capability. 

To overcome these limitations, the MicroElectroMechanical System (MEMS)-based sensor has been used in civil infrastructure monitoring, especially in bridge health monitoring [24,25,26,27]. However, few applications of MEMS-based sensors in pavement vibration monitoring reported in the literature. Bajwa et al. used a wireless sensor network for vehicle classification, weight estimation, and pavement performance monitoring. The Wireless Sensor Networks (WSN) includes acceleration sensors, magnetometer sensors, a camera, and an access point [28,29,30]. Ma et al. described a prototype axle count and spacing automatic vehicle classification system using wireless accelerometers and magnetometers [31]. Stocker et al. demonstrated the application of a pavement vibration sensor network system to vehicle detection and classification. They use the machine learning algorithm to process sensor data and acquire knowledge [32,33]. Denis Kleyko et al. applied the feature-free data smashing method to the problem of vehicle classification based on magnetometer and accelerometer measurements from roadside sensors [34]. They focus on the application of monitoring systems and the design of the algorithm, and they use commercial acceleration sensor nodes to obtain vibration data.

In this paper, an acceleration sensing node is developed by using a MEMS accelerometer, which can be desirably used in pavement vibration monitoring. More specifically, node component selection, low energy consumption hardware design, and node encapsulation can all be accomplished. Subsequently, water resistance and compressive tests of the sensing nodes were carried out. Sensing performance was also analyzed and compared. These tests can be used to ensure that the sensing nodes can be effectively used in a practical road environment. Finally, the acceleration sensing nodes were equipped for practical pavement vibration monitoring in the presence of moving loads of three types of vehicles. The vehicle speed, type, and traffic flow information can be estimated from the pavement vibration data by using effective and efficient algorithms. The effects of vehicle speed, vehicle weight, and d on acceleration amplitude are statistically evaluated. This paper can provide guidance for the design, layout optimization, and embedded algorithm development of an acceleration sensing node.

## 2. Objective

There are two objectives in this study. The first objective is to develop an acceleration sensing node based on the MEMS accelerometer that can monitor minor vibrations of pavement under a vehicle load. The second objective is to apply the acceleration sensing node to achieve traffic information monitoring. The effects of d, vehicle speed, and weight on the speed error, wheelbase error, and acceleration amplitude are analyzed. The developed methodology and results can be used to study the vehicle-induced pavement vibration response and further realize a smart road.

## 3. Node Development

**Figure 1:**
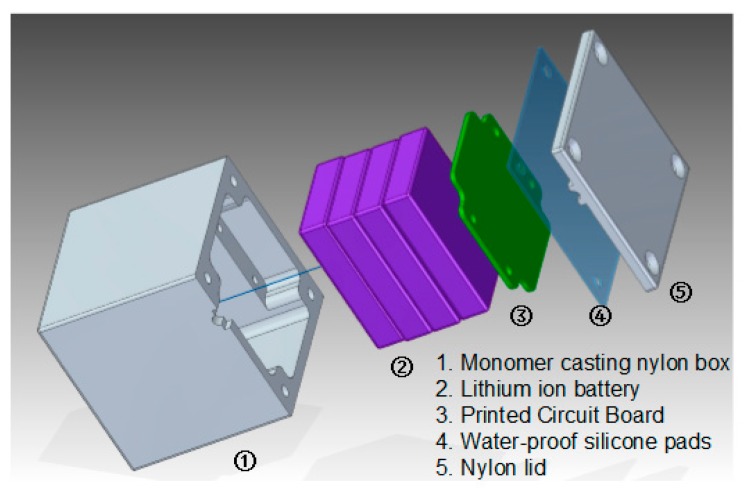
Acceleration sensing node.

**Figure 2:**
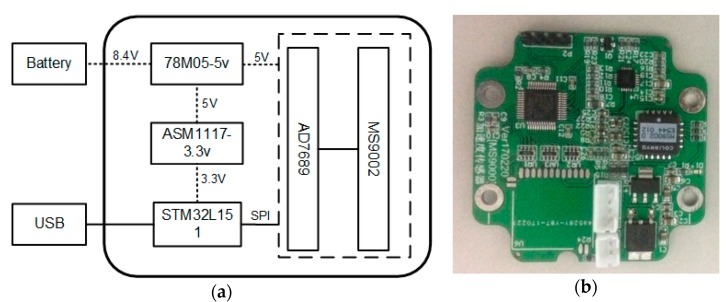
The printed circuit board (PCB) of the acceleration sensing node. (**a**) PCB composition; (**b**) PCB.

The acceleration sensing node consists of a monomer casting nylon box, lithium-ion battery, PCB (printed circuit board), waterproof silicone pad, and nylon lid, shown in Figure 1. The PCB consists of an ultra-low power CPU, MEMS acceleration sensor, analog to digital converter (ADC), and low dropout (LDO) regulator chip, shown in Figure 2.

The size of the acceleration sensing node is 6 cm × 6 cm × 6 cm, and monomer casting nylon is used as the external packaging material. The nylon box and waterproof silicone pad are used to make the node waterproof and dustproof, so that the PCB and battery are adequately protected. Four 3000 mAh lithium-ion batteries are used to generate 8.4 V voltage for the PCB.

The ADC (AD7689) can convert the output voltage signals of the MEMS acceleration sensor (MS9002) into digital signals. The digital signals will be output from the serial port after undergoing calculations and processing by the CPU (STM32L151). The power of the sensing node is provided by an 8.4 V lithium battery. Notably, the supply power of the sensor and ADC can be decreased from 8.4 V to 5 V by 78M05-5v, and CPU power can be decreased from 5 V to 3.3 V by ASM1117-3.3v. 

With appropriate component selection and hardware design, low energy consumption and high efficiency can be achieved for the acceleration sensing node, with the STM32L151 processor requiring 890 μA (4 MHz) in run mode and 9 μA (32 KHz) in low-power run mode. Moreover, the MS9002 costs less than 400 uA with 5 V power supply, and the AD7689 only requires 2.5 mA to achieve a 100 ksps sampling rate and 1 nA in standby mode. Therefore, the power consumption of the developed acceleration sensor node becomes significantly less than that of the piezoelectric acceleration sensor. The high energy costs of the piezoelectric acceleration sensor are due to the use of an adapter and a data acquisition device.

## 4. Performance Testing

### 4.1. Encapsulation Performance Testing

(1) Waterproof Testing

**Figure 3:**
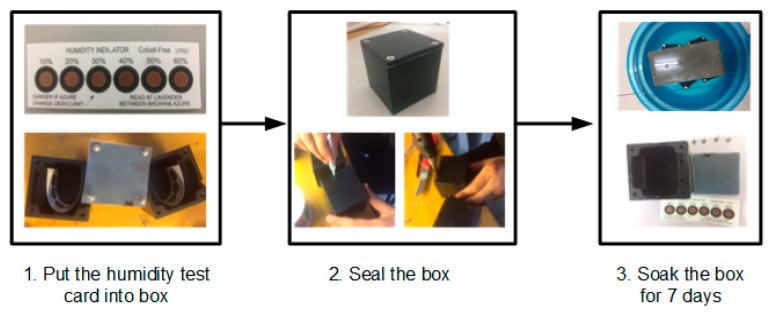
Waterproof testing.

According to the requirements of international industrial waterproofing standard IPX8, the procedures of a continuous diving test are presented in Figure 3.

Firstly, the humidity test card was placed in a nylon box. The top lid was screwed onto the nylon box. Subsequently, the silicone pad was strongly adhered to the nylon box and the outlet was blocked with hot-melt glue (epoxy). Finally, the nylon box was soaked in water; a brick was also used to prevent the box from floating. After soaking for a week, the box was opened and the humidity test card had not become blue, which indicated good waterproof performance of the sensing node encapsulation.

(2) Compressive Testing

**Figure 4:**
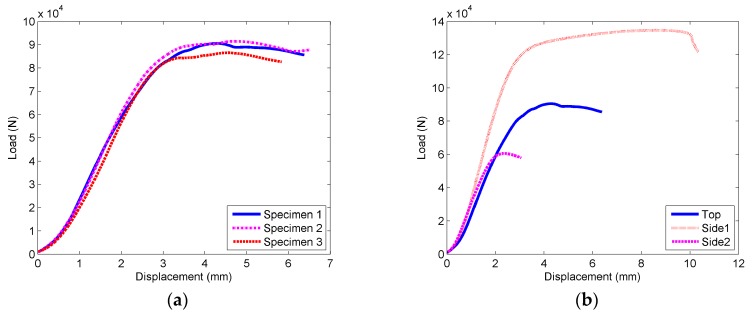
Load–displacement curves of the nylon box. (**a**) Compression of the front of the boxes; (**b**) Compression of different surfaces of the boxes.

By utilizing a universal testing machine, the nylon box was axially loaded at a loading rate of 2 mm/min. The compressive load–displacement curves of the specimen were obtained, as shown in Figure 4. The loading was terminated once the maximum compressive load was reached.

In Figure 4, the load–displacement curves can be divided into two separate stages. The first stage is defined as the linear elastic stage, which represents the stage before the initial compressional buckling of specimen. In this stage, the load–displacement curve is approximately linear. The second stage is defined as the yield stage, where the specimen begins to undergo wrinkle deformation. In this stage, the growth rate of compressive load can be significantly reduced. However, the compressive load still gradually increases until reaching the maximum load, where the specimen is bulging and deforming.

In Figure 4a, the load–displacement curves are shown for compression of the front of the boxes. The maximum yield pressures are 90.542 kN, 86.541 kN, and 91.434 kN, respectively, showing the ability to bear stress of at least 73.03 Mpa. In Figure 4b, the load–displacement curves are shown for the compression of different surfaces of the boxes, namely, Top, Side 1, and Side 2. The internal structure of the specimen could result in different pressure capacity for the different surfaces. The thickness of the supporting wall for Side 1 and Side 2 is 1.25 cm and 0.4 cm, respectively. It can be seen that the compressive strength prominently increases with the increase in the wall thickness. Notably, the maximum yield stress of Side 2 is still at 67.54 Mpa, which is far above the load of a vehicle [35]. 

The results show that the encapsulation of the acceleration sensing node is waterproof and can withstand the vehicle load. Therefore, the sensing nodes can be effectively applied to monitor pavement vibration in practice.

### 4.2. Sensing Performance Analysis

**Figure 5:**
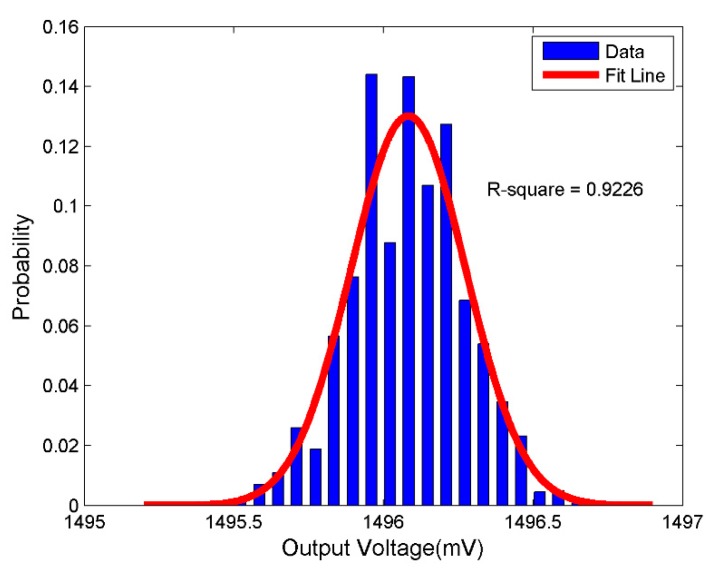
Probability density distribution of the output voltage when XOUT = −1 g.

It should be noted that a static calibration method was used to calibrate the acceleration sensing nodes. This can be performed with the known relationship between the output response of MS9002 and sensitive axis direction. Let us define the gravitational direction as the negative direction of the sensor sensitive axis. In this case, the output voltage corresponds to an acceleration value of −1 g. In Figure 5, the recorded output voltage is shown at the positive direction of the sensor sensitive axis (XOUT = −1 g). The output voltage fluctuates within ±1 mV, which can be considered as the sum of a constant value and noise value. The noise value can be approximately fitted using Gaussian distribution. Based on the minimum Root Mean Square (RMS), the constant value can be calculated by using the regression analysis technique.

**Figure 6:**
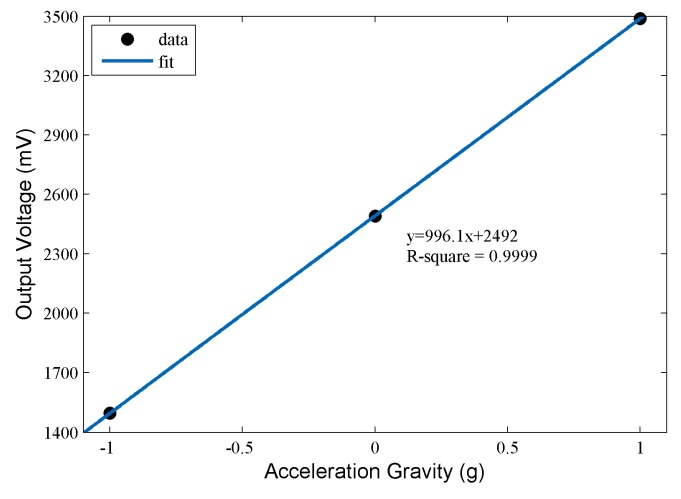
Relationship between output voltage and acceleration value.

The constant values at the negative direction and horizontal direction can be determined in a similar way. Finally, the relationship between the output voltage and the acceleration value can be obtained, which is shown in Figure 6.

According to the fitting result in Figure 6, the relationship between acceleration and output voltage can be considered to be approximately linear (*R*^2^ = 0.9999). This indicates good performance of the sensing node, and the sensitivity of the sensing node can be calculated as 966.1 mV/g. Moreover, the noise level of the sensing node is calculated as 0.198 mV in the horizontal direction. Therefore, the obtained resolution of the sensing node is 0.199 mg, which can completely satisfy the requirements of pavement vibration monitoring.

### 4.3. Comparison Test

**Figure 7:**
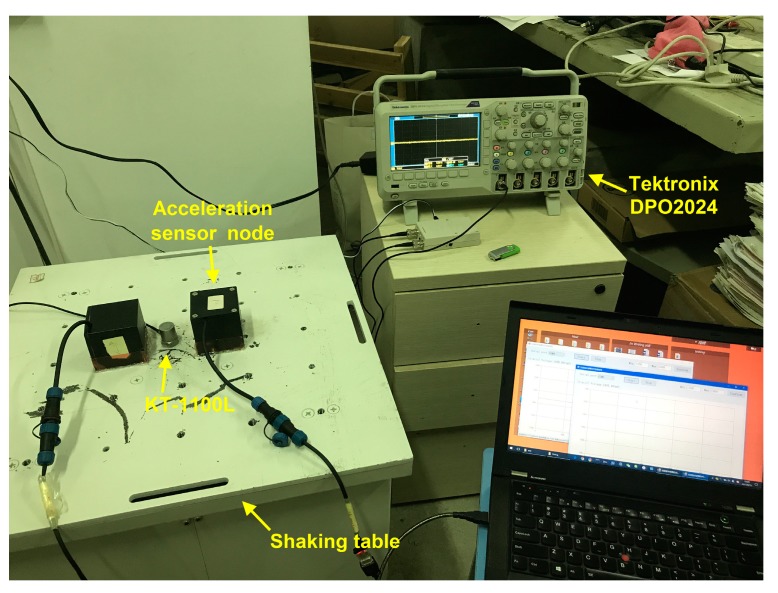
The comparison test.

In order to verify the performance of the developed acceleration sensing node, it is compared with the commercial piezoelectric accelerometer. The piezoelectric sensor KT-1100L is produced by Yangzhou KeTu Electronics Co., Ltd. in China, with a sensitivity of 1014 mV/g and a frequency test range of 0.5–5000 Hz. The Tektronix DPO2024 oscilloscope is used to acquire its voltage signals. The acceleration sensing nodes and KT-1100L accelerometer are fixed on a shaking table with hot-melt adhesive, as shown in Figure 7.

**Figure 8:**
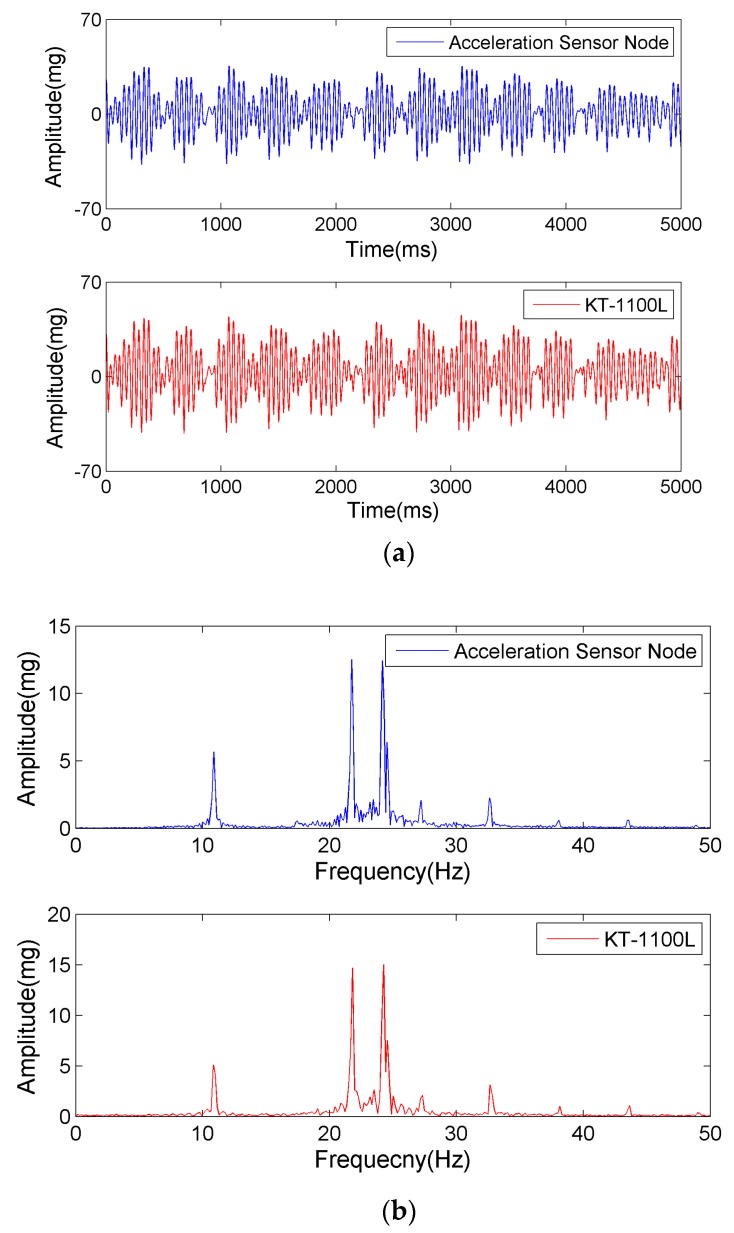
The measured acceleration signals on the shaking table. (**a**) Time domain signal; (**b**) Frequency domain signal.

Through random excitation to the shaking table, the acceleration sensors acquire multiple low-frequency vibration signals, which are shown in Figure 8.

Figure 8 shows that the output signals of the acceleration sensing node and piezoelectric sensor are close to each other in the time and frequency domains. This indicates that the developed acceleration sensing node can respond well to low-frequency vibration and recognize the frequency.

## 5. Field Tests

### 5.1. Experimental Scheme

**Figure 9:**
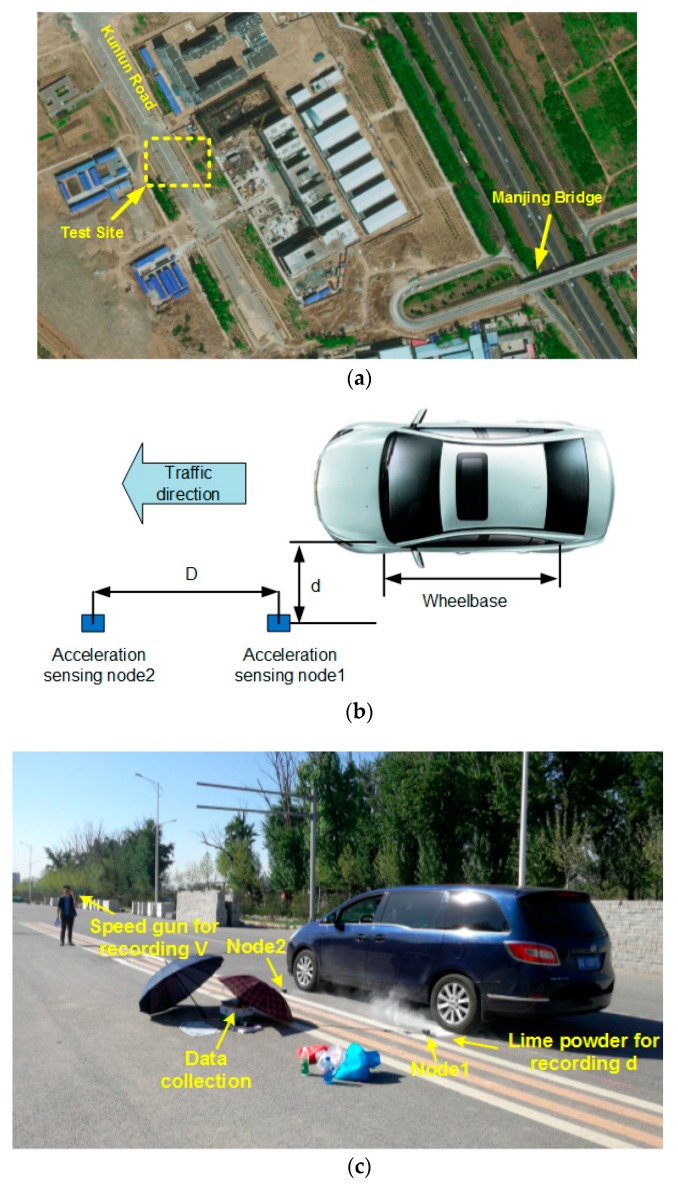
Field experiment. (**a**) Test site; (**b**) Test setup; (**c**) Field test.

The test site is chosen to be in the Kunlun Road, which is a semi-rigid base asphalt road near the Manjing Bridge, Beijing. The field experiment setting is shown in Figure 9.

As shown in Figure 9, two sensing nodes were spaced apart in the travelling direction, where the distance between two nodes was fixed as *D*. The distance between Node1 and the nearest tire center line was defined to be *d*. The vehicle speed was recorded using a radar speed gun, and *d* was measured by spreading lime powder to record the position of wheel trace. 

There are three types of vehicles passing by the monitoring points at several different speeds and values of *d*. The types of vehicles are summarized in Table 1.

### 5.2. Traffic Information Monitoring

(1) Speed and Wheelbase Estimate

**Figure 10:**
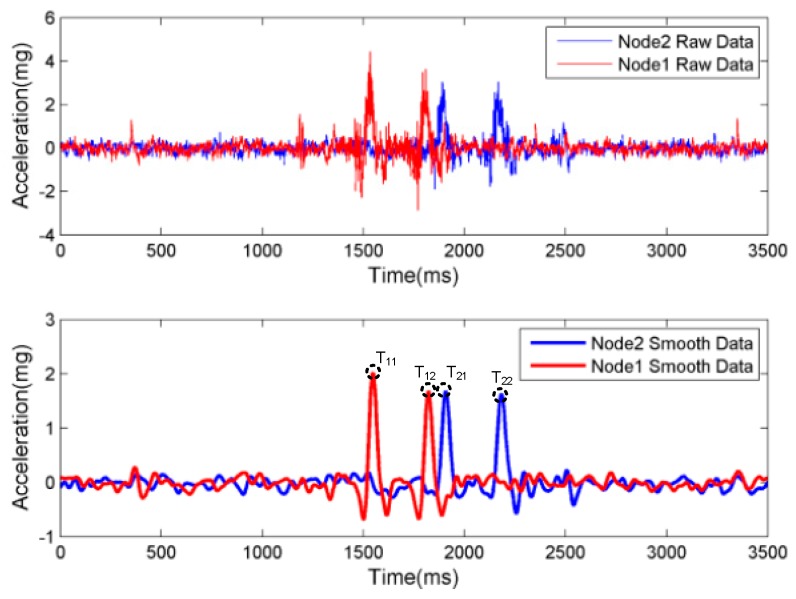
The pavement vibration response while a vehicle passes.

In Figure 10, the pavement vibration signals are shown for Node1 and Node2. It can be observed that the vibration generated by passing vehicles can be detected by using the sensing node in a quantitative manner. For the same monitoring point, there are two peaks which can be produced by front and back wheels of the vehicles. There exist time delays for the peaks at different monitoring points. The raw data could be smoothed by using the following formula [36], where the peaks and their corresponding time points could be identified:

(1) $$\bar{A}\left(t_{i}\right)=\;\frac{1}{B}\overset{+B}{\underset{-B}{\sum }}A\left(t_{i+j}\right)W_{j},$$

(2) $$W_{j}=\frac{15}{16}{\left(1-{\left(\frac{j}{B}\right)}^{2}\right)}^{2},$$

where $\bar{A}\left(t_{i}\right)$ and *A*(*t_i_*) represent the smoothed and measured acceleration at time *t_i_*, respectively; *B* is the smoothing bandwidth; and *W_j_* is the individual kernel weight for any discrete *j* with |*j*| ≤ *B*. The bandwidth was selected to be 30 ms.

To estimate the vehicle speed and wheelbase, the time corresponding to the first peak of Node1 and to the second peak of Node1 are defined as *T*_11_ and *T*_12_, respectively. Similarly, the two peaks corresponding to Node2 are denoted by *T*_21_ and *T*_22_. It is noted that the spacing distance between the two nodes is *D*. Therefore, the vehicle speed and wheelbase can be calculated as follows:

(3) $$V=D/\left(T_{21}-T_{11}\right),$$

(4) $$W=V\times \left(T_{12}-T_{11}\right).$$

The estimated values of speed and wheelbase could be obtained under different vehicle speed, vehicle types, and *d* value based on the above method; the results are shown in Table 2.

Based on the above method, the estimated speed and wheelbase are consistent with the true value as shown in Table 2. In other words, the acceleration sensing node can be applied to speed and wheelbase monitoring.

(2) Traffic Flow Monitoring

**Figure 11:**
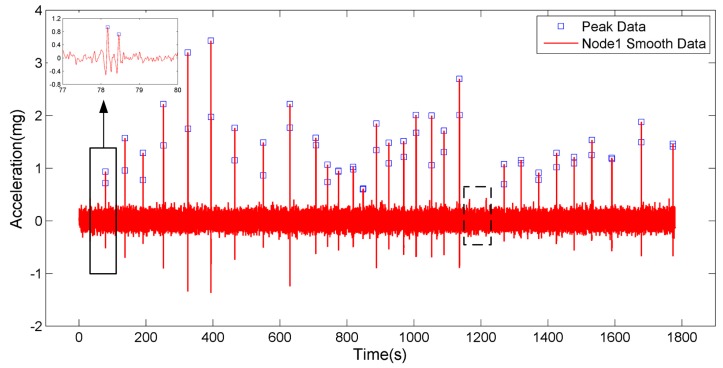
The smoothed data in a certain period of time.

The nodes are laid out as shown in Figure 9 and monitor pavement vibration in real time. The experimental vehicles pass through the monitoring area one by one, and the number of the passing vehicles is counted by the experimenter. The raw data of Node1 are smoothed in a certain period of time as shown in Figure 11.

Traffic flow monitoring can be performed by extraction of the peaks. Traffic flow can be monitored with the sum of the peaks from Node1, which can be expressed as

(5) $$SS=\;\sum \text{​}S_{i}$$

where *S_i_* represents the number of peaks obtained from the sensor Node1 installed in the road side. As an index of traffic flow, *SS* would change in correspondence with each axle of loads on the pavement. Twenty-four visible fluctuations of *SS* are clearly presented in Figure 11, which means there are 30 vehicles and 60 axles in total in the experiment. In fact, the total vehicles are 32. Two vehicles, which are indicated by the dashed box in Figure 11, are not detected. This is because the large *d* value or slow vehicle speed causes smaller peak values which are vulnerable to noise. If we use high-performance sensor nodes or deploy multiple sensor nodes in future practical applications, with this method, the numbers of axles and vehicles can be easily calculated at the same time for the traffic flow with desirably low calculation costs.

### 5.3. Error Analysis

To analyze the effects of *d* and vehicle speed on the speed error, wheelbase error, and acceleration amplitude, the vibration amplitude is defined, which is the difference between the maximum and minimum raw data values of the first fluctuation in the Node1. As shown in Figure 10, the amplitude of vibration is 6.729 mg.

(1) Controlled Vehicle Speed (*V* = 54–56 km/h) and Changed *d* Value

**Figure 12:**
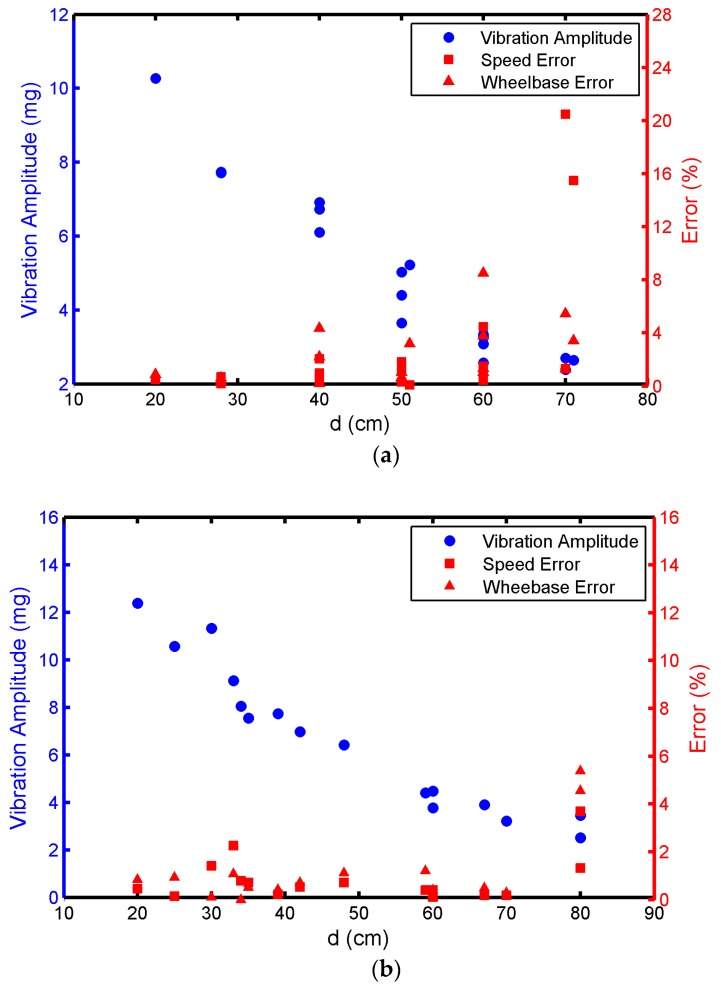

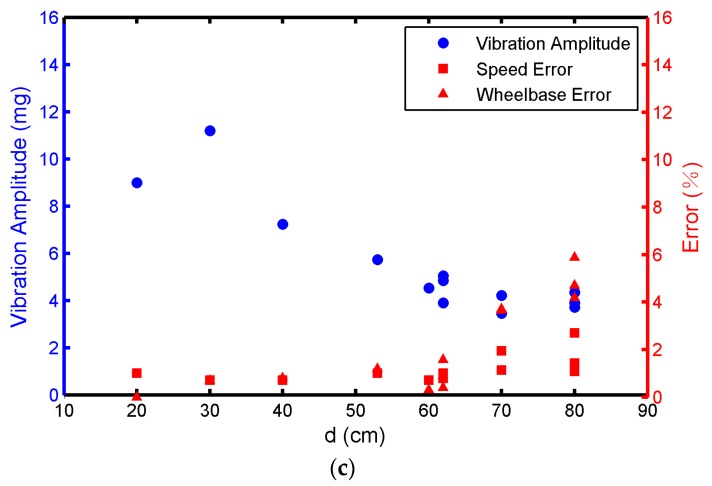
The effects of d on the vibration amplitude, speed error, and wheelbase error. (**a**) Buick GL-8; (**b**) Ford Transit; (**c**) Toyota Coaster.

In Figure 12a, the vehicle speed error is basically controlled within 2% if the vehicle is a GL-8, *d* is within 50 cm, and the wheelbase error is controlled within 5%. If *d* is larger than 50 cm, the error can significantly increase with the increase in *d*. If *d* becomes 70 cm, the acceleration sensing node is not able to estimate the vehicle speed and wheelbase accurately. Figure 12b,c show that the speed error and wheelbase error are controlled within 2% when the vehicle is a Ford Transit or Toyota Coaster and *d* is within 70 cm. The errors become even larger when *d* is larger than 70 cm.

(2) The *d* Value (*d* = 28–33 cm) Is Controlled and Vehicle Speed Is Changed

**Figure 13:**
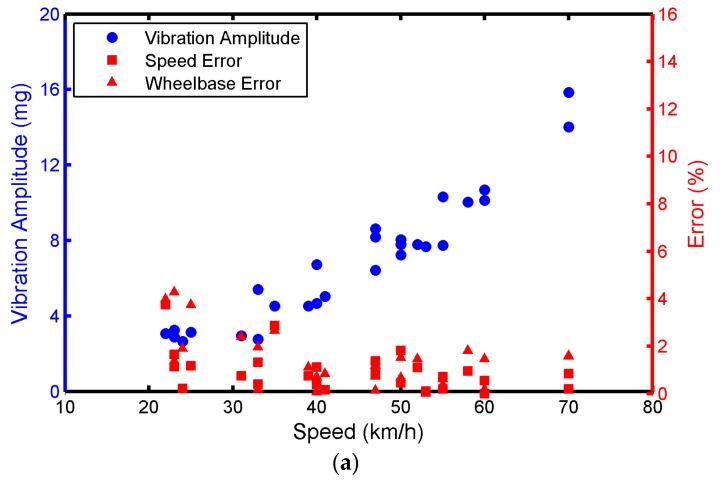

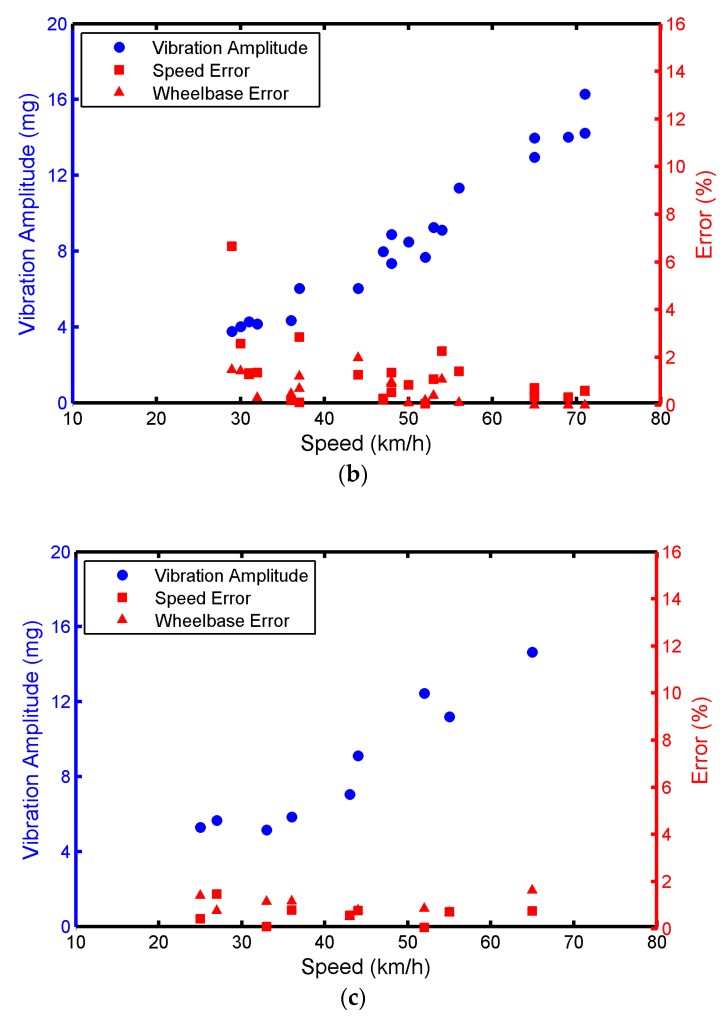
The effect of vehicle speed on the vibration amplitude, speed error, and wheelbase error. (**a**) Buick GL-8; (**b**) Ford Transit; (**c**) Toyota Coaster.

Figure 13a,b show that the error is generally less than 2% when the vehicle is a GL-8 or Ford Transit and the vehicle speed is greater than 40 kmh. If the vehicle speed is less than or equal to 30 kmh, the error can increase with the decrease in the speed. In Figure 13c, the error can be fixed within 2% when the vehicle is a Toyota Coaster and the speed is 25–30 km/h, due to the large weight of the Coaster. Moreover, vibration amplitude can decrease with the increase in *d* when the speed is controlled in the range 54–56 km/h. When *d* is controlled in the range 28–33 cm, vibration amplitude can increase with the increase in the speed. The vibration amplitude is also positively correlated with vehicle weight.

(3) Correlation and Variance Analysis

Correlation and variance analysis were used to analyze the effects of different weights, speeds, and *d* values on vibration amplitude, speed error, and wheelbase error, which is shown in Table 3 and Table 4.

In Table 3, it can be observed that the vibration amplitude is positively correlated with vehicle weight and speed and negatively correlated with *d*. The speed error is negatively correlated with weight and positively correlated with *d*. The wheelbase error is positively correlated with *d*. It should also be noted that speed and *d* have a significant impact on vibration amplitude. There is no direct correlation of speed with speed error and wheelbase error.

In Table 4, if the vibration amplitude becomes larger than 3.5 mg, the average errors of speed and wheelbase are 1.11% and 1.32%, respectively. When the vibration amplitude is smaller than 3.5 mg, the error becomes prominently larger. The reason for this is that smaller vibration amplitude will make the sensing node more susceptible to noise and the environment. Therefore, it becomes challenging to distinguish the characteristic of voltage fluctuations. To obtain accurate monitoring, the vibration amplitude should be larger than 3.5 mg, which can be affected by the vehicle speed, vehicle weight, and the loading position.

## 6. Conclusions

In this paper, a low energy consumption acceleration sensing node is developed for traffic information monitoring, including vehicle speed, wheelbase, and traffic flow. In particular, the main contribution can be summarized as follows.

(1) Node component selection, hardware design, and node encapsulation are accomplished. The waterproof and compression tests are carried out and sensing performance is analyzed. These tests have validated that the sensing nodes can be applied in the real road environment.
(2) The acceleration sensing node is applied to practical pavement vibration monitoring. Efficient algorithms have also been proposed to process the vibration data generated by three types of vehicles. The vehicle speed, vehicle type, and traffic flow can be accurately estimated.
(3) The vibration amplitude is positively correlated with vehicle weight and speed and negatively correlated with *d*. The average errors of speed and wheelbase are 1.11% and 1.32%, respectively, when the vibration amplitude becomes larger than 3.5 mg. The errors of speed and wheelbase become prominently larger when the vibration amplitude is smaller than 3.5 mg, which can be set as a threshold value to obtain accurate monitoring.
(4) For future work, acceleration sensing nodes can be fitted with a wireless transmission module to transmit the vibration data higher than a threshold value. Therefore, the communication power consumption can be reduced and remote monitoring can be facilitated. Besides this, multiple sensing nodes will be deployed optimally to monitor traffic and infrastructure information under various environmental and loading conditions.

## Figures and Tables

**Table 1:** Vehicle Type.

Name	Passenger Capacity	Wheelbase (mm)	Curb Weight (kg)
Buick GL-8	7	3088	1840
Ford Transit	15–17	3750	2170–2400
Toyota Coaster	23	3935	3466–3694

**Table 2:** Vehicle speed and wheelbase estimates.

Pass	Vehicle Types	Vehicle Offset *D* (cm)	Speed (km/h)		Wheelbase (mm)	
			Speed Gun	Estimated Value	Actual Value	Estimated Value
1	Buick GL-8	20	54	54.27	3088	3060.3
2		32	41	41.06		3114.07
3		40	38	37.63		3041.45
4		50	53	52.68		3087.8
5		25	60	60.34		3117.32
6	Ford Transit	23	29	28.76	3750	3786.95
7		35	54	54.38		3731.12
8		45	30	30.5		3889.82
9		47	64	64.29		3714.29
10		60	53	52.79		3753.67
11	Toyota Coaster	20	32	31.8	3935	3910.1
12		30	43	42.76		3954.87
13		40	53	52.63		3903.51
14		50	64	64.06		3950.18
15		60	55	55.38		3923.08

**Table 3:** Correlation analysis.

Variable	Vibration Amplitude	Speed Error	Wheelbase Error
Weight	0.265 **	−0.140 *	−0.27
Speed	0.824 **	−0.110	−0.28
d	−0.849 **	0.349 **	0.264 **

Note: ** *p* < 0.001; * *p* < 0.05.

**Table 4:** Variance analysis.

Variable	Vibration Amplitude <3.5 mg		Vibration Amplitude >3.5 mg		F	P
	Average	Standard Deviation	Average	Standard Deviation		
Speed error	3.82	6.063	1.11	1.142	38.649	<0.001
Wheelbase error	3.55	7.428	1.32	1.225	17.777	<0.001
